# Effect of SOD2 methylation on mitochondrial DNA4834-bp deletion mutation in marginal cells under oxidative stress

**DOI:** 10.17305/bjbms.2019.4353

**Published:** 2020-02

**Authors:** Jun Li, Xiang Dai, Xuelian He, Rong Yang, Zhongfang Xia, Han Xiao

**Affiliations:** 1 Department of Otorhinolaryngology, Wuhan Children’s Hospital, Tongji Medical College, Huazhong University of Science and Technology, Wuhan, China; 2 Laboratory of Reproduction, Wuhan Children’s Hospital, Tongji Medical College, Huazhong University of Science and Technology, Wuhan, China; 3 Central Laboratory, Wuhan Children’s Hospital, Tongji Medical College, Huazhong University of Science and Technology, Wuhan, China; 4 Department of Health, Wuhan Children’s Hospital, Tongji Medical College, Huazhong University of Science and Technology, Wuhan, China; 5 Biological Sample Bank, Wuhan Children’s Hospital, Tongji Medical College, Huazhong University of Science and Technology, Wuhan, China

**Keywords:** Oxidative stress, superoxide dismutase 2, SOD2, methylation, mtDNA4834 deletion, age-related hearing loss

## Abstract

Presbycusis, or age-related hearing loss, is a prevalent disease that severely affects the physical and mental health of the elderly. Oxidative stress and mitochondrial (mt)DNA deletion mutation are considered as major factors in the pathophysiology of age-related hearing loss. The 4977-bp deletion in human mtDNA (common deletion, corresponding to the 4834-bp mtDNA deletion in rats) is suggested to be closely associated with the pathogenesis of age-related hearing loss. Superoxide dismutase 2 (SOD2), an isoform of SOD that is exclusively expressed in the intracellular mitochondrial matrix, plays a crucial role in oxidative resistance against mitochondrial superoxide. Previous research has shown that methylation of the promoter region of the *SOD2* gene decreased the expression of *SOD2* in marginal cells (MCs) extracted from the inner ear of rats subjected to D-galactose-induced mtDNA4834 deletion. However, the relationship between *SOD2* methylation and mtDNA4834 deletion under oxidative stress remains to be elucidated. Herein, an oxidative damage model was established in the extracted MCs using hydrogen peroxide (H_2_O_2_), which increased the methylation level of *SOD2* and the copy number of mtDNA4834 mutation in MCs. Decreasing the methylation level of *SOD2* using 5-azacytidine, a DNA methylation inhibitor, reduced oxidative stress and the copy number of mtDNA4834 mutation and inhibited H_2_O_2_-induced apoptosis. The present work demonstrates that decreasing the methylation of *SOD2* suppresses the mtDNA4834 deletion in MCs under oxidative stress and provides potential insights to the intervention therapy of aging-related hearing loss.

## INTRODUCTION

Presbycusis or aging-related hearing loss is a prevalent disease in the elderly, most often defined as a progressive process of bilateral and symmetrical sensorineural hearing loss in response to age-associated degeneration of inner ear structures. More than 300 million people worldwide suffer from presbycusis, and this number is predicted to reach 900 million by 2050 [1]. Hearing deficiency has been suggested to be closely associated with cognitive impairment, depression, and social isolation of elderly people [2], emphasizing the importance of presbycusis treatment.

Oxidative stress increases during the aging process, leading to increased reactive oxygen species (ROS) production and lowered antioxidant defense [3]. Numerous investigations have revealed the relationship between oxidative stress and aging-related conditions such as skeletal muscle function decline [4], Alzheimer’s disease [5], macular degeneration [6], cardiovascular diseases [3], and diabetes [7]. Oxidative stress was also considered to play a crucial role in the pathophysiology of age-related hearing loss [8]. For example, inhibiting the process of oxidative stress alleviated hearing loss in D-galactose-induced aging mice [9]. Additionally, oxidative stress can induce mitochondrial DNA (mtDNA) deletion mutation, which is considered as another major factor in the pathophysiology of aging-related hearing loss [8]. A previous study has demonstrated that high levels of ROS induced the mtDNA4977 deletion, resulting in peptic ulcer disease [10]. The 4977-bp deletion in human mtDNA (corresponding to the 4834-bp mtDNA deletion in rats) is a common deletion. The occurence of the mtDNA4977 deletion in the archival cochlear tissue and celloidin-embedded temporal bone sections of patients experiencing aging-related hearing loss suggested the involvement of the mtDNA4977 deletion in its pathogenesis [11,12].

Superoxide dismutase 2 (SOD2), an isoform of SOD that is exclusively expressed in the intracellular mitochondrial matrix [13], plays a crucial role in oxidative defense against mitochondrial superoxide [14,15]. SOD2 deficiency has been suggested to increase the level of mtDNA mutation damage [16,17]. High levels of ROS in the aging process lead to the methylation of *SOD2*, which in turn decreases the transcription level of *SOD2* [18]. Our preliminary experiments have shown that the methylation of the promoter region of the *SOD2* gene decreased the expression of SOD2 in marginal cells (MCs) extracted from the inner ear of rats subjected to D-galactose-induced mtDNA4834 deletion (not shown). In addition, oxidative damage to MCs has been considered as an important factor in the pathogenesis of sensorineural deafness [19,20]. However, the relationship between *SOD2* methylation and mtDNA4834 deletion under oxidative stress remains to be elucidated.

In this work, MCs were treated with hydrogen peroxide (H_2_O_2_) to establish an oxidative damage model as previously described [21]. H_2_O_2_ decreased the expression of *SOD2* by increasing the methylation level of *SOD2*, which was attenuated by 5-azacytidine (AZA), a DNA methylation inhibitor. Also, the copy number of mtDNA4834 deletion in MCs was detected to investigate the effect of *SOD2* methylation on the mtDNA4834 deletion in MCs under oxidative stress.

## MATERIALS AND METHODS

### MC extraction and treatment

As reported previously [21], Wistar rats (0–3 days old, supplied by the Laboratory Animal Centre, Huazhong Agricultural University) were anesthetized using pentobarbital sodium (Sigma, MO, USA) and sacrificed by cervical dislocation. Bilateral auditory vesicles were obtained and immersed in D-Hanks’s solution. Cochlear stria vascularis were removed under a microscope and evenly cut into pieces (7–10 pieces/cochlea). The pieces were placed into a Petri dish and digested with 0.1% collagenase II for 30 min, followed by centrifugation for 5 min at 1000 rpm and resuspension in serum-free MEM-α (Hyclone, Utah, USA) containing 2 mmol/l of L-glutamine (Gibco, Grand Island, NY, USA) and 1% penicillin-streptomycin-amphotericin B solution (Bioswamp, Myhalic Biotechnology Co., Ltd., Wuhan, China) for 1 h in a polylysine-coated 6-well plate. Finally, the obtained cells were incubated in serum-free MEM-α containing 10% fetal bovine serum at 37°C in an atmosphere containing 5% CO_2_. Dead and non-adherent cells were removed by refreshing the culture medium after 24h of culture. The medium was refreshed twice a week. Cell morphology was observed under a microscope (Nikon, Tokyo, Japan). When the MCs reached approximately 90% confluence, they were seeded into a 96-well plate (5 × 10^3^ cell/well) and cultured for 24 h. The medium was replaced and H_2_O_2_ was added at different concentrations (200, 300, 400, 600, and 800 µmol/l), followed by 0.5, 1, 2, 4, 16, or 24 h of culture. After further incubation for 24 h with culture medium, the cell viability was detected using the 3-(4,5-dimethyl-2-thiazolyl)-2,5-diphenyl-2-H-tetrazolium bromide (MTT) assay to select the optimal concentration and time for the establishment of the oxidative damage model. Then, the cells were divided into three groups: control (untreated, denoted as CON), H_2_O_2_ (treated with H_2_O_2_ alone, denoted as H_2_O_2_), and H_2_O_2_ plus AZA (treated with H_2_O_2_ and 0.25 µmol/l AZA, denoted as H_2_O_2_ + AZA).

### MTT assay

After the MCs were treated, 20 µl of MTT reagent (Bioswamp) was added to each well and the cells were incubated for 4 h at 37°C in an atmosphere containing 5% CO_2_. The supernatant was removed and 150 µl of dimethyl sulfoxide was added to each well. After 10 min of low-speed shaking, the absorbance of each well was measured using a microplate reader (Thermo, Waltham, MA, USA). All experiments were performed in triplicate.

### Bisulfite sequencing polymerase chain reaction (BSP)

BSP was performed to evaluate the methylation status of *SOD2*. A TIANamp Genomic DNA kit (Tiangen Biotech Co., Ltd., Beijing, China) was used to extract genomic DNA, and a DNA Bisulfite Conversion Kit (Tiangen Biotech) was used for bisulfite conversion. The converted DNA (75 ng) was then subjected to amplification using a T100-Thermal Cycler apparatus (Bio-Rad, USA) with the following reaction procedure: 95°C for 5 min; 30 cycles of denaturation at 95°C for 10 s, annealing at 56°C for 10 s, and extension at 72°C for 10 s; and final extension at 72°C for 5 min. The SOD2 primers were as follows: forward, 5’-TAAGTGAGTTAGAAGGATTTTGA-3’ and reverse, 5’-TATACTCCACCCTCAAACTAAACC-3’. All experiments were performed in triplicate.

### Quantitative reverse transcription polymerase chain reaction (qRT-PCR)

Total RNA was extracted from MCs using Trizol (Ambion, TX, USA), followed by reverse transcription into cDNA using the M-MuLV kit (TaKaRa, Dalian, China). cDNA (25 ng) was subjected to amplification with the following *SOD2* primer sequences: forward, 5’-ATTGCCGC CTGC TCTA-3’ and reverse, 5’-CTCCCAGTTGAT TACATTC C-3’. Glyceraldehyde 3-phosphate dehydrogenase [GAPDH] (forward, 5’-CAAGTTCAACGGCACAG-3’ and reverse, 5’-CCAGTAGACTCCACGACAT-3’) served as an internal control. The 2^-∆∆Ct^ method was used to calculate the relative mRNA expression levels [22].

### Immunofluorescence

The expression of SOD2 in MCs was detected using immunofluorescence. MCs were fixed using 4% paraformaldehyde for 30 min at room temperature and immersed in 0.5% Triton X-100 (Bioswamp) for 20 min. Thereafter, the cells were blocked using 5% bovine serum albumin for 1 h at 37°C and incubated with primary antibodies against SOD2 (Abcam, ab13533, 1:100) overnight in a humidified chamber at 4°C and Alexa Fluor 594-conjugated goat anti-rabbit secondary antibody (Bioswamp, PAB160018, 1:200) for 30 min in a humidified chamber at 37°C. The cell nuclei were stained using 4’,6-diamidino-2-phenylindole [DAPI] (Bioswamp), and the expression of SOD2 was detected using an inverted fluorescence microscope.

### Biochemistry test

The expression of nitric oxide (NO) and SOD2 activity in the supernatant of extracted MCs was examined by a colorimetric method according to the manufacturer’s instruction (NO: A013-2; SOD2: A001-1-2; Nanjing Jiancheng Bioengineering Institute, Nanjing, China).

### Flow cytometry

Flow cytometry was performed to detect ROS production and apoptosis. For the intracellular ROS assay, the collected cells (1 × 10^7^ cell/ml) were mixed with diluted 2’,7’-dichlorofluorescin diacetate (DCFH-DA) fluoroprobes (Bioswamp, 10 µmol/l) for 20 min at 37°C with gentle shaking every 4 min. After uncontacted DCFH-DA was eliminated by washing with serum-free medium, the cells were detected using a NovoCyte^TM^ apparatus (ACEA Biosciences, San Diego, CA, USA). To evaluate apoptosis, the Annexin V-FITC/propidium iodide (PI) assay (BD, Shanghai, China) was carried out according to the manufacturer’s protocol. Harvested cells (1 × 10^5^) were resuspended in 200 µl of binding buffer (BD), followed by incubation with 10 µl of Annexin V-FITC and 10 µl of PI in the dark for 30 min at 4°C. Thereafter, the cells were subjected to flow cytometry.

### Western blot

The expression of apoptosis-related proteins caspase 3, B-cell lymphoma-2 (Bcl-2), Bcl-2-associated x (Bax), and cytochrome C (Cyt-c) was detected using Western blot assay. Total protein content was extracted using radioimmunoprecipitation assay lysis buffer (Bioswamp) supplemented with protease and phosphatase inhibitors. A bicinchoninic acid kit (Bioswamp) was used for protein quantification. Proteins (20 µg) were separated by sodium dodecyl sulfate-polyacrylamide gel electrophoresis and transferred onto polyvinylidene fluoride membranes (Millipore, MA, USA). After blocking with 5% skim milk, the membranes were incubated with primary antibodies against caspase 3 (Abcam, ab13847, 1:1000), cleaved caspase 3 (Abcam, ab2302, 1:1000), Bcl-2 (Abcam, ab196495, 1:1000), Bax (Abcam, ab182733, 1:2000), Cyt-c (Abcam, ab133504, 1:5000), and GAPDH (CST, 2118, 1:1000) overnight at 4°C, followed by incubation with goat anti-rabbit immunoglobulin (Ig)G secondary antibody (Bioswamp, PAB150011, 1:10000) for 1 h at room temperature. Immunoreactivity was visualized by colorimetric reaction using an enhanced chemiluminescence substrate buffer (Millipore, MA, USA). The membranes were then detected using a Tanon-5200 apparatus (Tanon Science & Technology Co., Ltd., Shanghai, China) and the band gray values were read.

### MtDNA4834 mutation copy number detection

The copy number of mtDNA4834 mutation was detected using qRT-PCR with the following mtDNA4834 mutation primer sequences: forward, 5’-GAACCTGAGCCCTAATAAT-3’ and reverse, 5’-GATAGCTGAGTGGTAGGGG-3’ as mentioned above. The equation of the standard curve is:

y = -0.48x + 27.081

where y represents the CT value and x represents the copy number.

### Statistical analysis

The data are presented as the mean ± standard deviation (SD). Differences between groups were analyzed using one-way analysis of variance. A value of *p* < 0.05 was considered to be statistically significant.

## RESULTS

### Damaging effect of H_2_O_2_ on MCs

**FIGURE 1:**
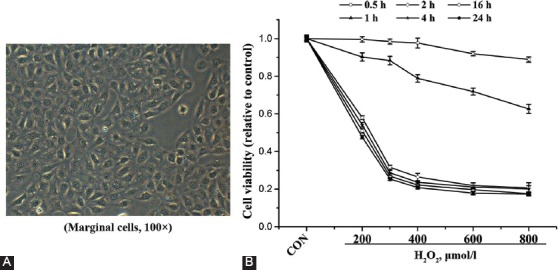
Effect of hydrogen peroxide (H_2_O_2_) on marginal cells (MCs). (A) Morphology of MCs. (B) Viability of MCs after treatment with H_2_O_2_ at different concentrations and for different time periods. Data represent mean ± standard deviation [SD] (n = 3).

Cells observed under a conventional microscope showed typical morphology of a pleomorphic growth pattern and clear boundaries, suggesting the successful isolation of MCs (Figure 1A). The isolated MCs were then treated with H_2_O_2_ and the cell viability was detected using an MTT assay. The results demonstrated that H_2_O_2_ decreased the viability of MCs in a time- and dose-dependent manner within a certain range (Figure 1B). Since lethal dose 50 (LD50) value of H_2_O_2_ for MCs is 200 µmol/l for 2 h, MCs treated with H_2_O_2_ at a concentration of 200 µmol/l for 2 h were selected for the subsequent experiments.

### Inhibition of *SOD2* methylation restored the H_2_O_2_-induced decrease of *SOD2* expression in MCs

**FIGURE 2:**
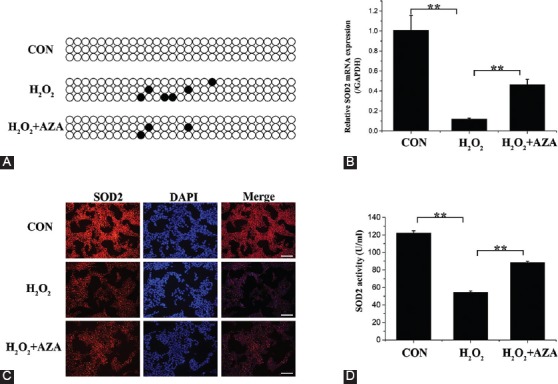
Characterization of superoxide dismutase 2 (SOD2). (A) Bisulfite sequencing polymerase chain reaction (BSP) of the methylation of *SOD2* gene promoter. Methylated and unmethylated sites are shown as black and white dots, respectively. (B) Relative mRNA expression of *SOD2*. (C) Immunofluorescence of SOD2. (D) SOD2 activity. Data represent mean ± standard deviation [SD] (n = 3). **Denotes p < 0.01. Scale bar = 100 µm.

After MCs were treated with H_2_O_2_ with or without AZA, the methylation of *SOD2* was evaluated using BSP. Compared to control cells, H_2_O_2_ promoted the methylation of the *SOD2* promoter, which was suppressed by AZA (Figure 2A). Furthermore, the mRNA expression of *SOD2* (Figure 2B) was significantly decreased by H_2_O_2_ (*p* < 0.01) but subsequently increased by AZA (*p* < 0.01). Immunofluorescence staining suggested that the positive expression of SOD2 was decreased in the H_2_O_2_ group compared to that in the CON group (Figure 2C). Additionally, H_2_O_2_ suppressed the expression of SOD2 in the supernatant (*p* < 0.01), which was significantly increased after combination treatment with AZA (*p* < 0.01, Figure 2D). Taken together, H_2_O_2_ increased the methylation level of *SOD2* in MCs, in turn suppressing the protein expression of SOD2, and this phenomenon was counteracted by AZA through decreasing the methylation level of *SOD2*.

### Inhibition of *SOD2* methylation reduced H_2_O_2_-induced oxidative stress in MCs

**FIGURE 3:**
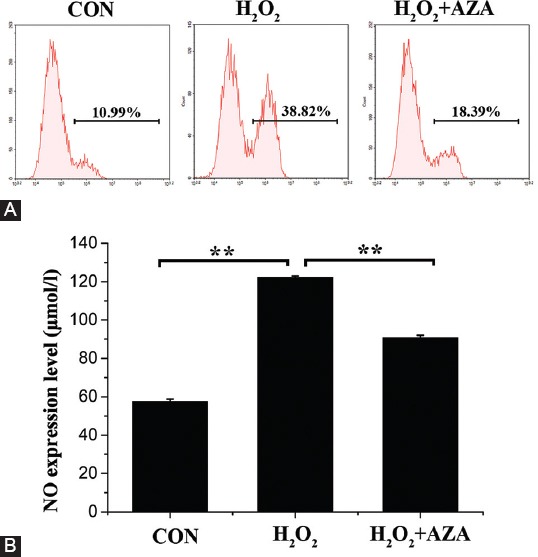
Effect of superoxide dismutase 2 (SOD2) methylation on hydrogen peroxide (H_2_O_2_)-induced oxidative stress of marginal cells (MCs). (A) Reactive oxygen species (ROS) production and (B) nitric oxide (NO) levels. Data represent mean ± standard deviation [SD] (n = 3). **Denotes p < 0.01.

Flow cytometry was carried out to detect ROS production in MCs. As shown in Figure 3A, the proportion of normal MCs exhibiting upregulated ROS production was approximately 10.99% and increased to 38.82% after H_2_O_2_ treatment. In the presence of AZA, the proportion of ROS-producing cells decreased to 18.39% after H_2_O_2_ treatment. In particular, compared to the CON group, the expression of NO in the H_2_O_2_ group was notably increased (*p* < 0.01). Compared to the H_2_O_2_ group, the expression of NO in the H_2_O_2_ + AZA group was obviously decreased (*p* < 0.01; Figure 3B). The results demonstrated that inhibition of *SOD2* methylation attenuated H_2_O_2_-induced oxidative stress by decreasing ROS generation and NO activity.

### Inhibition of *SOD2* methylation attenuated H_2_O_2_-induced mtDNA4834 deletion

**FIGURE 4:**
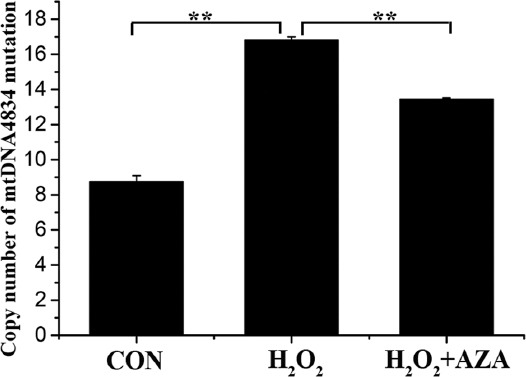
mtDNA4834 mutation copy number. Data represent mean ± standard deviation [SD] (n = 3). **Denotes p < 0.01.

As shown in Figure 4, compared to untreated MCs, the copy number of mtDNA4834 mutation was increased after H_2_O_2_ treatment compared to that in untreated MCs (*p* < 0.01) and decreased in the presence of AZA (*p* < 0.01). These results demonstrated that inhibition of *SOD2* methylation attenuated H_2_O_2_-induced mtDNA4834 deletion.

### Inhibition of *SOD2* methylation attenuated H_2_O_2_-induced apoptosis

**FIGURE 5:**
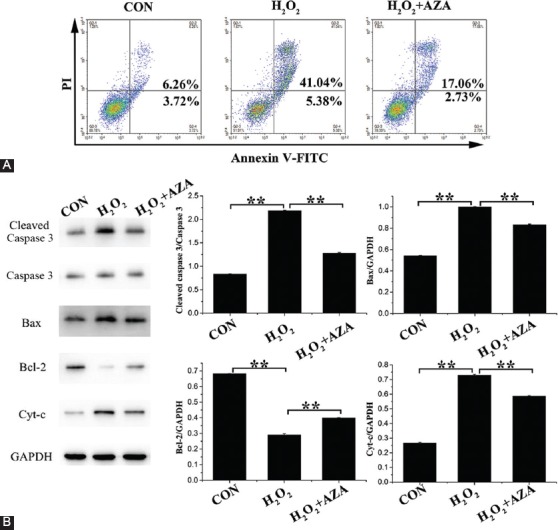
Effect of superoxide dismutase 2 (*SOD2*) methylation on hydrogen peroxide (H_2_O_2_)-induced apoptosis of marginal cells (MCs). (A) MCs apoptosis rate. (B) Expression and quantification of apoptosis-related proteins in MCs. Data represent mean ± standard deviation [SD] (n = 3). **Denotes p < 0.01.

The apoptosis of MCs treated with H_2_O_2_ with or without AZA was examined using flow cytometry. Compared to untreated MCs, apoptosis increased by 36.44% after MCs were treated with H_2_O_2_. Compared to H_2_O_2_ treatment alone, the addition of AZA decreased MC apoptosis by 26.63% (Figure 5A). Next, apoptosis-related proteins were detected using Western blot (Figure 5B). The results suggest that the expression of the pro-apoptosis proteins cleaved caspase 3, Bax, and Cyt-c was increased after H_2_O_2_ treatment compared to that in untreated MCs, but the co-treatment with AZA reduced their expression. Meanwhile, the expression of anti-apoptosis Bcl-2 showed the opposite tendency. These results indicate that inhibition of *SOD2* methylation attenuated H_2_O_2_-induced apoptosis.

## DISCUSSION

Our data provide the first evidence demonstrating that inhibition of *SOD2* methylation suppresses the mtDNA4834 deletion mutation in MCs under oxidative stress. Oxidative stress induced by H_2_O_2_ increases the methylation level of *SOD2*, in turn reducing the expression of SOD2. AZA treatment decreased the methylation of *SOD2*, inhibited H_2_O_2_-induced mtDNA4834 deletion mutation, and attenuated H_2_O_2_-induced oxidative stress, as demonstrated by the decrease in ROS and NO levels.

Aging is a complex process that is widely accepted to be associated with oxidative stress [23]. The progressive degeneration of physiological function is suggested to be the result of the accumulation of oxidative damage caused by ROS [24]. Mitochondria are a major site of oxidative damage, including protein, lipid, and mtDNA damage [24,25]. Oxidative stress-induced mtDNA damage is correlated with a variety of aging-related diseases, such as cataract [26], amyotrophic lateral sclerosis [27], and macular degeneration [28]. In addition, oxidative stress-induced mtDNA deletion mutation is associated with the pathogenesis of aging-related hearing loss in the auditory cortex of the central auditory system of rats [29]. The findings of this study demonstrated that H_2_O_2_-induced oxidative stress increased the deletion of mtDNA4834 in MCs. SOD2, a key factor of aging and defense against oxidative stress, is exclusively located in the extracellular and intracellular mitochondrial matrix [13,30] and plays a crucial role in the oxidant resistance against mitochondrial superoxide [14,15]. As the first line of defense against mitochondrial oxidative damage, SOD2 is the major enzyme that scavenges ROS in the mitochondrial matrix [31]. However, high levels of ROS in the aging process lead to the methylation of *SOD2* DNA, in turn decreasing the transcription level of *SOD2* [18] and resulting in the failure of oxidative damage defense. This work demonstrated that H_2_O_2_-induced oxidative stress promoted the methylation of *SOD2*, thus inhibiting SOD2 transcription. Inhibition of *SOD2* methylation increased the transcription level of *SOD2*, thus attenuating oxidative stress as demonstrated by the decrease in ROS and NO levels. In addition, inhibition of *SOD2* methylation decreased the extent of mtDNA4834 deletion caused by H_2_O_2_-induced oxidative stress.

An increasing body of evidence in animal experiments has demonstrated that oxidative stress in the cochlea results in mtDNA deletion and impaired mitochondrial function, thus inducing cochlear cell apoptosis and promoting the development of aging-related hearing loss [32]. Mitochondrial apoptosis, also termed endogenous apoptosis, is characterized by the release of caspase activators such as Cyt-c into the cytoplasm, resulting in a series of cascade reactions [33,34]. This process is related to changes in the permeabilization of the outer mitochondrial membrane induced by proteins from the Bcl-2 family [35,36]. This work demonstrated that oxidative stress contributed to mitochondrial apoptosis of MCs by upregulating the expression of Cyt-c, in turn activating caspase 3. Furthermore, the expression of the pro-apoptosis protein Bax and anti-apoptosis protein Bcl-2 was regulated by the oxidative stress-induced methylation of *SOD2*, which might be mediated by the decrease in mtDNA4834 deletion.

## CONCLUSION

Our study demonstrated that oxidative stress increased the methylation level of *SOD2* and mtDNA4834 deletion in MCs, which was suppressed by inhibiting the methylation of *SOD2*. The inhibition of *SOD2* methylation attenuated MC oxidative stress-induced apoptosis by regulating mitochondrial apoptosis-related proteins. The present work provides potential insight into intervention therapy against aging-related hearing loss.
